# Prevalence, characteristics and outcomes of respiratory viral co-infection among hospitalized children

**DOI:** 10.1007/s00431-026-07056-5

**Published:** 2026-05-12

**Authors:** Iddo Reisler, Yoel Levinsky, Osnat Tausky, Doron Mulla, Bar Goldberg, Noam Itzhaki-Wygoda, Oded Scheuerman, Lotem Goldberg

**Affiliations:** 1 Department of Pediatrics B, Schneider Children’s Medical Center of Israel, 14 Kaplan Street, 4920235 Petah Tiqva, Israel; 2 Gray Faculty of Medical and Health Sciences, Tel Aviv University, 69978 Tel Aviv, Israel; 3 Data Research Center, Research Authority, Schneider Children’s Medical Center of Israel, 4920235 Petah Tiqva, Israel

**Keywords:** Respiratory tract infections, Viral co-infection, Inflammatory markers, Pediatric hospitalizations

## Abstract

Respiratory tract infections (RTIs) are a leading cause of pediatric hospitalization. Although multiple concurrent viral infections are frequently detected, their impact on clinical outcomes remains unclear. This study evaluated whether viral co-infection in children leads to worse clinical outcomes, higher inflammatory markers, or increased medical management compared to single-virus infections. We retrospectively studied children aged 0–18 years hospitalized with RTIs at a tertiary pediatric center (2017–2024). Inclusion required at least one virus detected by multiplex polymerase chain reaction (PCR). Clinical, laboratory, and hospitalization data were compared between children with single versus multiple viral infections. Multivariable regression models adjusted for age, sex, and season were used to assess associations with clinical outcomes. Of 5,703 hospitalized children, 1,120 (19.6%) had multiple viral infections. Co-infected children were younger (median 0.9 years [IQR 0.5—1.6] vs. 1.2 [IQR 0.4—3.2], *p* < 0.0001) and more likely to have elevated inflammatory markers, including higher C-reactive protein (CRP) and white blood cell (WBC) counts. Despite these differences, key hospitalization outcomes, including length of stay (median 4 days [IQR 3–6] in both groups), intensive care unit (ICU) admission (8.0% vs. 7.2%), and 30-day rehospitalization rates, did not differ significantly. Co-infected patients were more frequently treated with bronchodilators and steroids.

*Conclusions*: Viral co-infections were common, particularly among younger children, and were associated with modestly higher inflammatory responses and increased use of anti-inflammatory medications. However, co-infection did not significantly affect hospitalization duration or ICU admission. These findings suggest that multiple viral infections may not substantially worsen disease severity in hospitalized children.

**Table:** 

**What is Known:** • *Viral co-infections are common in pediatric respiratory illness, yet their impact on clinical severity and outcomes remains debated and inconsistent in current literature.*
**What is New:** • *Co-infection is associated with significantly higher inflammatory markers and increased use of steroids and bronchodilators compared to single-virus infections.* • *Despite laboratory differences and higher medication use, co-infection does not increase length of stay, ICU admission rates, or 30-day rehospitalization.*

**What is Known:**

• *Viral co-infections are common in pediatric respiratory illness, yet their impact on clinical severity and outcomes remains debated and inconsistent in current literature.*

**What is New:**

• *Co-infection is associated with significantly higher inflammatory markers and increased use of steroids and bronchodilators compared to single-virus infections.*

• *Despite laboratory differences and higher medication use, co-infection does not increase length of stay, ICU admission rates, or 30-day rehospitalization.*

## Introduction

Respiratory tract infections (RTIs) are among the most common illnesses in children [1]. They present with a wide clinical spectrum, ranging from mild upper respiratory symptoms to severe, life-threatening lower respiratory tract disease [2].

The majority of RTIs are viral in origin, most often caused by pathogens such as respiratory syncytial virus (RSV), influenza virus, parainfluenza virus, human metapneumovirus (hMPV), and adenovirus [3].

The prevalence of respiratory tract infections (RTIs) caused by more than one virus simultaneously in children has been reported to range from less than 1% to over 40%. Current literature offers no clear consensus on the impact of viral co-infections on clinical outcomes [4, 5]. Multiple studies have compared children with a single identified respiratory virus to those with two or more concurrent viral pathogens. However, these studies often suffer from important limitations, including a focus on specific virus pairs [6, 7], a focus on young infants [6, 8], and the use of outdated diagnostic techniques, such as viral culture rather than polymerase chain reaction (PCR) [8, 9]. Moreover, their findings are inconsistent: while some report that co-infections are associated with a milder clinical course, others suggest they contribute to increased disease severity. Understanding how multiple viral infections affect children with respiratory illness is essential to improving diagnosis and treatment.

In this study, we aim to evaluate whether hospitalized children with multiple concurrent viral infections experience different clinical outcomes compared to those with a single identified respiratory pathogen. Leveraging a large cohort of 5,703 children across the full pediatric age spectrum, we provide a comprehensive analysis of laboratory indices and medical management associated with viral co-infection.

## Methods

### Study design and setting

This was a retrospective study conducted at Schneider Children's Medical Center, a tertiary university affiliated, medical center in Israel. The study was approved by the Research Ethics Board of Rabin Medical Center (Approval No. RMC 0272–22) in accordance with the Declaration of Helsinki.

### Participants

Children aged 0 to 18 years hospitalized in the pediatric wards between 2017 and 2024 were eligible for inclusion. Only those who underwent respiratory viral panel testing during hospitalization and had at least one respiratory virus detected from a nasopharyngeal swab were included. The study population was categorized into two study groups based on the viral exposure: a single-virus group and a multiple-virus group (defined as the detection of two or more concurrent pathogens).

### Data collection

Clinical data were extracted from electronic medical records and included demographics and underlying medical conditions. The primary clinical outcomes of the study were defined as hospitalization length of stay, ICU admission, and 30-day rehospitalization rates. Secondary outcomes included clinical parameters, laboratory and microbiology results, diagnostic imaging rates, and medical management (use of antibiotics, bronchodilators, and steroids). For variables with missing data, a complete-case analysis approach was used.

### Sample collection and viral detection

Nasopharyngeal swab samples collected during routine clinical care were analyzed. Viral detection was performed using multiplex real-time polymerase chain reaction (PCR) assays targeting common respiratory viruses, including respiratory syncytial virus (RSV), influenza viruses, parainfluenza viruses, human metapneumovirus, adenovirus, rhinovirus, and SARS-CoV-2 (COVID-19). Prior to 2019, viral testing was conducted using in-house PCR assays. From 2019 to 2023, the *Allplex™ RV Essential Assay* (Seegene, South Korea) was utilized, and since 2023, testing has been performed with the *Allplex™ RV Master Assay* (Seegene, South Korea). Testing for rhinovirus was integrated into the diagnostic panels starting in 2020. Between 2020 and 2023, SARS-CoV-2 testing was conducted according to evolving sampling protocols, and has been performed for all patients tested for respiratory viruses since 2023.

### Statistical methods

Statistical analysis was performed using R software.

For patients with multiple hospitalizations during the study period, only the first hospitalization was included in the analysis to avoid duplication and ensure independence of observations.

Descriptive statistics were used to summarize baseline characteristics and study variables, both overall and stratified by study groups. Continuous variables were reported as means with standard deviations (SD), or as medians with interquartile ranges (IQR) for variables that were not normally distributed, while categorical variables were presented as counts and percentages. Group comparisons were performed using the Wilcoxon rank-sum test for continuous variables and the chi-square test for categorical variables.

Continuous outcomes were analyzed using linear regression models, and binary outcomes were assessed using logistic regression. Count outcomes (length of hospital stay, ICU hospitalization length, days of fever and days of oxygen desaturation) were truncated and transformed into ordinal categories, which were then analyzed using ordinal regression models. Maximal recorded fever values were rounded down to the nearest whole number and analyzed with ordinal regression as well; fever values exceeding 41.5°C were excluded to minimize the influence of outliers.

All regression models included the study group as the primary independent variable and were adjusted for sex, age group, and season (defined according to Israel’s climate).

## Results

### Study population

A total of 5,703 hospitalized children with one or more respiratory viral infections were included in the study. Among them, 4,583 (80.4%) had a single viral infection, while 1,120 (19.6%) were infected with two or more viruses concurrently (Table 1).

**Table 1:** Characteristics of study cohort

Variable	Children with single respiratory virus, *N* = 4,583 (80%) ^1^	Children with multiple viruses, *N* = 1,120 (20%) ^1^	Overall, *N* = 5,703^1^	*p*-value^2^
Sex				0.1169
Female	2,038 (44.5%)	469 (41.9%)	2,507 (44.0%)	
Male	2,545 (55.5%)	651 (58.1%)	3,196 (56.0%)	
Age (years)	1.2 (0.4—3.2)	0.9 (0.5—1.6)	1.1 (0.4—2.7)	<.0001
Age Group				<.0001
0—1	2,149 (46.9%)	620 (55.4%)	2,769 (48.6%)	
1—2	879 (19.2%)	310 (27.7%)	1,189 (20.8%)	
2 +	1,555 (33.9%)	190 (17.0%)	1,745 (30.6%)	
Season				0.0311
Winter	2,034 (44.4%)	485 (43.3%)	2,519 (44.2%)	
Spring	990 (21.6%)	209 (18.7%)	1,199 (21.0%)	
Summer	751 (16.4%)	215 (19.2%)	966 (16.9%)	
Fall	808 (17.6%)	211 (18.8%)	1,019 (17.9%)	
Congenital Heart Disease	125 (2.7%)	28 (2.5%)	153 (2.7%)	0.6728
Pulmonary Disease	150 (3.3%)	30 (2.7%)	180 (3.2%)	0.3077
Oncology Patient	119 (2.6%)	13 (1.2%)	132 (2.3%)	0.0042
Premature Birth	46 (1.0%)	12 (1.1%)	58 (1.0%)	0.8395
Primary Immunodeficiency	51 (1.1%)	7 (0.6%)	58 (1.0%)	0.1447
Solid Organ Transplant Recipient	45 (1.0%)	8 (0.7%)	53 (0.9%)	0.4028

^1^n (%) is used for categorical variables and median (IQR) is used for continuous variables

^2^Pearson’s Chi-squared test is used for categorical variables and Wilcoxon rank sum test is used for continuous variables

**Fig. 1:**
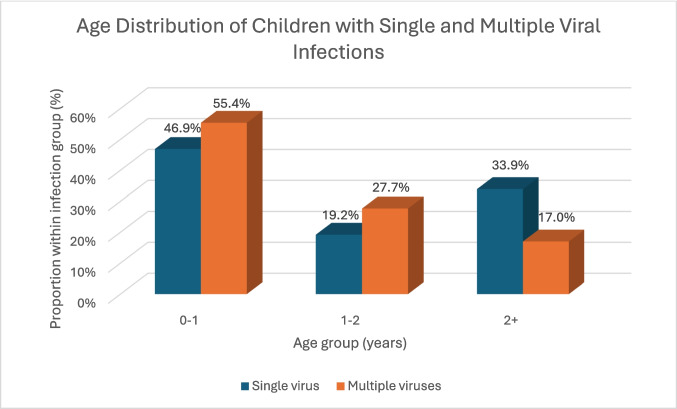
Age distribution of children with single and multiple respiratory viral infections. *p* <.0001 (Pearson’s Chi-squared test)

The median age of children in the study was 1.1 years (interquartile ranges [IQR] 0.4—2.7). Children with a single viral infection were significantly older (median 1.2 years, IQR 0.4—3.2) than those with multiple viral infections (median 0.9 years, IQR 0.5—1.6, *p* < 0.0001). The distribution of age groups by infection type is shown in Fig. 1, indicating that multiple viral infections were more common among younger children.

Regarding background medical conditions, 2.6% of patients in the single virus group were oncology patients, compared with 1.2% in the multiple viruses group (*p* = 0.0042). Other underlying conditions, including heart disease, pulmonary disease (comprising various conditions such as asthma, wheezing, and congenital anomalies), prematurity, primary immunodeficiency, and solid organ transplantation, were similarly distributed between the two groups. Within the pulmonary category, specific conditions included tracheostomy (*n* = 6) and cystic fibrosis (*n* = 2), all of which occurred in the single virus group. Additionally, of the 12 patients with tracheoesophageal fistula, 9 were in the single virus group and 3 were in the multiple viruses group.

### Viral detection

**Fig. 2:**
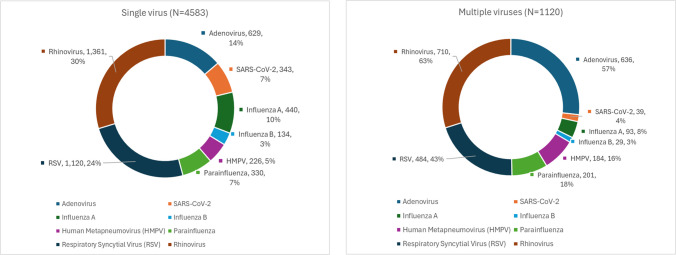
Distribution of respiratory viruses in single and multiple viral infections. Prevalence of individual respiratory viruses is shown for patients with single-virus infections (*N* = 4,583) and those with multiple-virus infections (*N* = 1,120). Data labels indicate the absolute number of cases and the corresponding percentage within each group

**Fig. 3:**
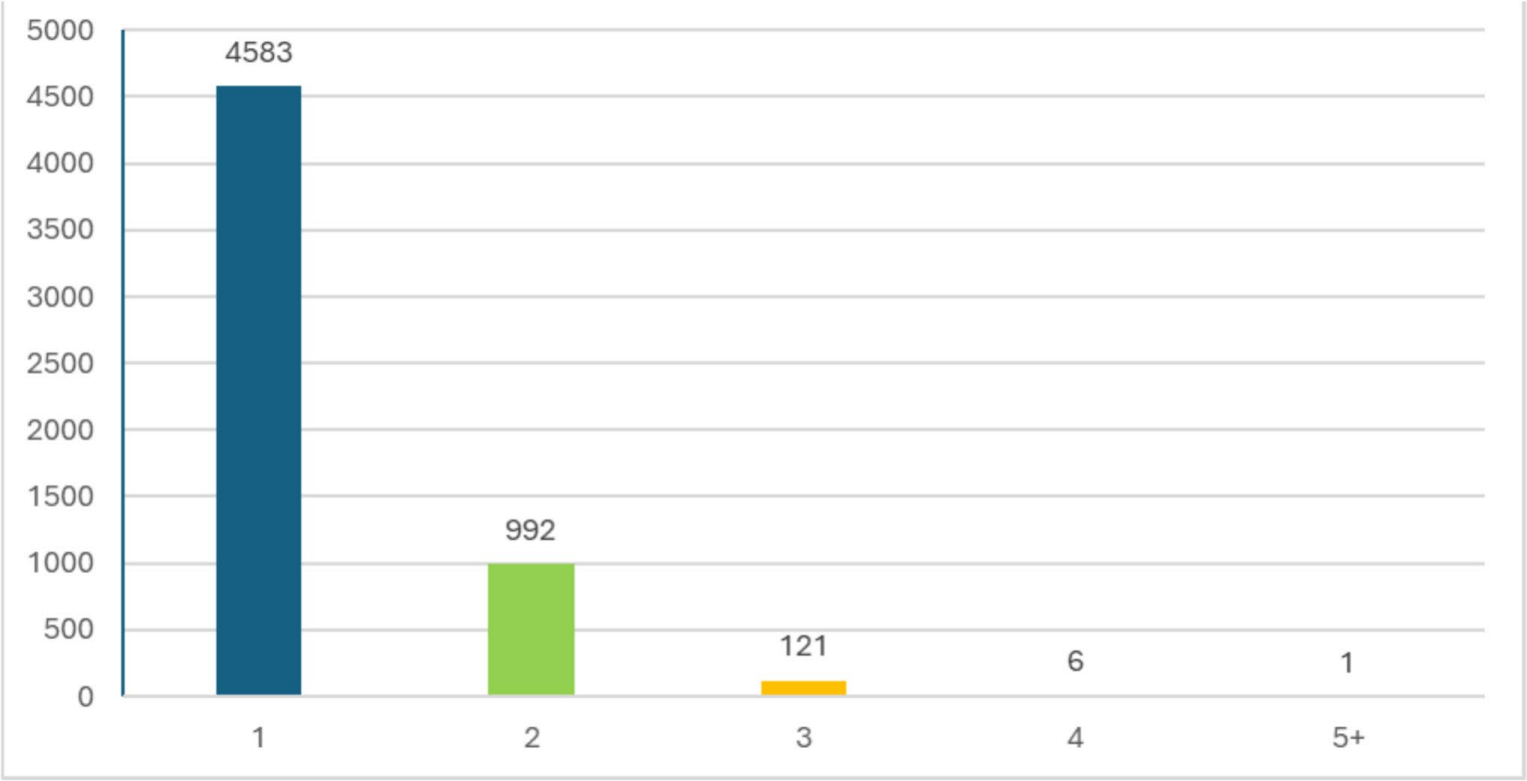
Frequency of single and multiple viral detections. The chart displays the number of distinct viruses identified across the study cohort. The majority of cases involved a single virus, with a decreasing frequency of co-infections involving two or more pathogens

In the single virus group, the most common viruses identified were rhinovirus (29.7%), respiratory syncytial virus (RSV) (24.4%), adenovirus (13.7%), influenza A (9.6%), and parainfluenza (7.2%) (Fig. 2). Among patients with co-infections, the detection of two viruses was most frequent (992 cases, 88.6%), followed by three viruses (121 cases, 10.8%) and four viruses (6 cases, 0.5%), with one rare case involving five concurrent viruses (0.1%) (Fig. 3). The predominant viruses in this group were rhinovirus (63.4%), adenovirus (56.8%), and RSV (43.2%), followed by parainfluenza (17.9%) and human metapneumovirus (16.4%). Overall, rhinovirus was the most commonly detected virus (36.3%), followed by RSV (28.1%) and adenovirus (22.2%).

### Hospitalization outcomes

The median length of stay was similar between groups, with 4 days (IQR 3—6) in both the single virus and multiple viruses groups (odds ratio [OR] 0.976, 95% confidence interval [CI] 0.869—1.097) (Table 2). ICU admission rates were also comparable, occurring in 7.2% of patients with a single virus and 8.0% of those with co-infections (OR 1.148; 95% CI 0.893—1.462; *p* = 0.273). The duration of ICU stay did not differ significantly between groups (median 3.5 [IQR 3—7.8] vs. 3 [IQR 2—7] days). Rehospitalization within 7 days was rare (0.2% in both groups), and rehospitalization within 30 days occurred in 2.3% of patients in each group, with no significant differences observed. Overall, no hospitalization outcomes showed a statistically significant association with viral co-infection.

**Table 2:** Hospitalization outcomes, clinical signs, and treatments in children with single vs. multiple respiratory viral infections

Variable	Children with single respiratory virus, *N* = 4,583^1^	Children with multiple viruses, *N* = 1,120^1^	Overall, *N* = 5,703^1^	OR^1^	95% CI^1^	*p*-value
Hospitalization results						
Hospitalization length (days)	4 [3–6]	4 [3–6]	4 [3–6]	0.976	0.869, 1.097	0.6844
ICU hospitalizations	329 (7.2%)	90 (8.0%)	419 (7.3%)	1.148	0.893, 1.462	0.2728
ICU hospitalization length (days)	3 [2–7]	3.5 [3—7.8]	3 [2–7]	1.190	0.775, 1.833	0.4267
Rehospitalization within 7 days	8 (0.2%)	2 (0.2%)	10 (0.2%)	1.052	0.159, 4.208	0.9491
Rehospitalization within 30 days	105 (2.3%)	26 (2.3%)	131 (2.3%)	1.148	0.724, 1.758	0.5393
Clinical Signs						
Maximal fever (N = 3644)	38.5 (1.06)	38.7 (1.03)	38.5 (1.05)	1.22	1.05, 1.42	0.0015
Days of fever above 38°C (N = 3644)				1.03	0.83, 1.26	0.0744
0 (afebrile)	1,020 (22.3%)	204 (18.2%)	1,224 (21.5%)			
1	751 (16.4%)	203 (18.1%)	954 (16.7%)			
2	556 (12.1%)	142 (12.7%)	698 (12.2%)			
3	264 (5.8%)	73 (6.5%)	337 (5.9%)			
4 +	355 (7.7%)	84 (7.5%)	439 (7.7%)			
Days of oxygen saturation under 90%	0.5 (1.62)	0.7 (2.25)	0.6 (1.76)	1.22	1.04, 1.42	0.0002
Positive blood culture	193 (4.2%)	51 (4.6%)	244 (4.3%)	1.20	0.87, 1.65	0.2572
Chest radiography performed	2,339 (51%)	619 (55.3%)	2,958 (51.9%)	1.22	1.07, 1.39	0.0034
Medications						
Antibiotics	1,802 (39.3%)	405 (36.2%)	2,207 (38.7%)	0.909	0.792, 1.043	0.1762
Bronchodilators	708 (15.4%)	220 (19.6%)	928 (16.3%)	1.350	1.135, 1.600	0.0006
Inhaled steroids	1,025 (22.4%)	347 (31.0%)	1,372 (24.1%)	1.511	1.303, 1.749	<.0001
Systemic steroids	991 (21.6%)	278 (24.8%)	1,269 (22.3%)	1.288	1.099, 1.507	0.0017

^1^Mean (SD); Median [IQR]; *n* (%), *OR* Odds Ratio, *CI* Confidence Interval

### Clinical signs

Patients with multiple viral infections had a slightly higher maximal fever compared to those with a single virus (mean 38.7°C vs. 38.5°C; OR 1.22; 95% CI 1.05—1.42). Fever duration showed a similar distribution between groups. Oxygen desaturation episodes (cumulative number of calendar days during which at least one documented oxygen saturation measurement was below 90%) were infrequent but slightly longer in co-infected patients (mean 0.7 vs. 0.5 days; OR 1.22; 95% CI 1.04—1.42). Overall, co-infection was associated with marginally higher fever and longer hypoxemia duration, though absolute differences were small.

### Laboratory indices

**Fig. 4:**
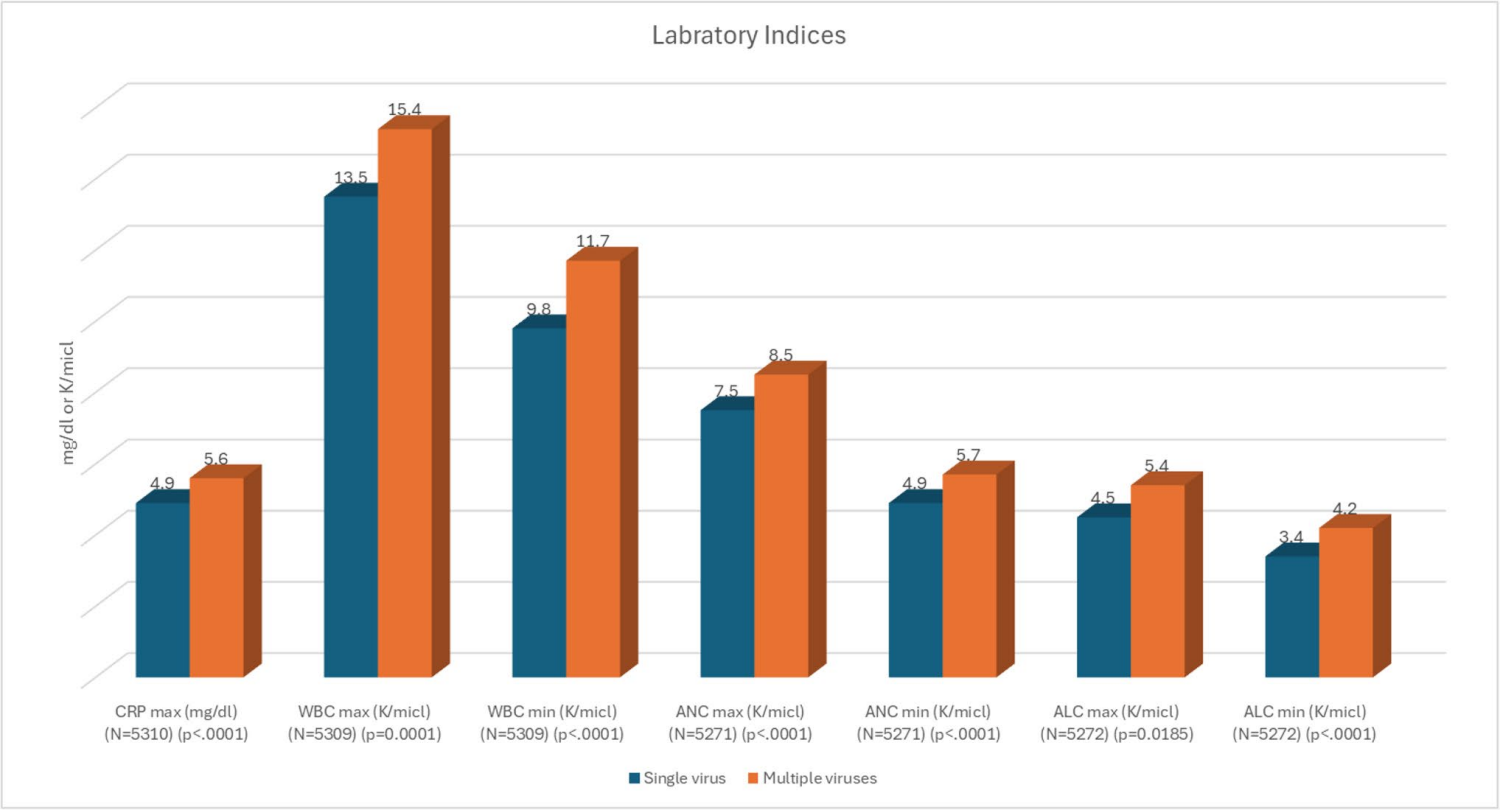
Laboratory findings in children with single vs. multiple respiratory viral infections. Data represent the mean values for each laboratory index. The sample size (N) for each specific parameter, is indicated on the x-axis. p-values were calculated using the Wilcoxon rank-sum test. Abbreviations: CRP: C-reactive protein, WBC: White blood cell count, ANC: Absolute neutrophil count, ALC: Absolute lymphocyte count

Patients with multiple viral infections showed higher inflammatory and hematologic indices compared to those with a single virus. Maximal CRP levels were modestly but significantly higher in the multiple-virus group (mean 5.6 mg/dL vs. 4.9 mg/dL, β = 1.06, 95% CI 0.58—1.54, *p* < 0.0001). Similarly, maximal and minimal WBC counts were elevated in the multiple-virus group. Children with multiple viral infections exhibited higher maximal absolute neutrophil counts and higher maximal absolute lymphocyte counts compared to the single-virus group. Detailed laboratory results are presented in Fig. 4. The proportion of positive blood cultures was similar between groups (4.6% vs. 4.2%, *p* = 0.26).

### Medications

Medication use differed between children with single and multiple viral infections. Antibiotic use was similar between groups (39.3% vs. 36.2%, *p* = 0.18). In contrast, children with multiple viral infections were more likely to receive bronchodilators (19.6% vs. 15.4%, OR 1.35, 95% CI 1.14—1.60, *p* = 0.0006) and both inhaled (31.0% vs. 22.4%, OR 1.51, 95% CI 1.30—1.75, *p* < 0.0001) and systemic steroids (24.8% vs. 21.6%, OR 1.29, 95% CI 1.10—1.51, *p* = 0.0017).

### Imaging

Children with multiple viral infections were more likely to undergo chest radiography evaluation compared with those with a single viral infection (55.3% vs. 51.0%, OR 1.22, 95% CI 1.07—1.39, *p* = 0.0034).

### Subgroup analysis by virus type

To evaluate the specific impact of co-infection, we performed a subgroup analysis comparing patients with a single viral detection to those with the same virus and at least one additional pathogen (Table 3). This analysis assessed WBC counts, CRP levels, length of stay (LOS), and ICU admission rates across individual viral subgroups. The overall trend of heightened inflammatory markers in co-infected patients was preserved in several subgroups. Specifically, maximal WBC levels were significantly higher in the co-infection group for RSV, rhinovirus, influenza A and B, HMPV, and SARS-CoV-2. Similarly, maximal CRP levels were significantly higher in co-infections involving RSV and influenza B. Notably, Adenovirus represented an exception, with significantly higher CRP levels observed in the single-infection group compared to co-infections. In contrast, length of stay and ICU admission rates did not show a consistent trend across viral subgroups.

**Table 3:** Clinical outcomes across viral subgroups: comparison between single and multiple infections

Virus	Total *N*	Single *N*	Multiple *N*	Max WBC (10^3^/mcL), mean ± SD (single vs. multiple)	*P*-value	Max CRP (mg/dL), mean ± SD (single vs. multiple)	*P*-value	LOS (days), median [IQR] (single vs. multiple)	*P*-value	ICU admission (%), single vs. multiple	*P*-value
RSV	1604	1120	484	12.9 ± 5.9 vs. 15.0 ± 7.2	** < 0.001**	3.7 ± 5.5 vs. 4.9 ± 6.5	** < 0.001**	4 [3–6] vs. 4 [3–6]	0.968	6.2% vs. 7.6%	0.323
Rhinovirus	2071	1361	710	14.7 ± 10.3 vs. 16.1 ± 10.4	** < 0.001**	5.7 ± 8.1 vs. 5.9 ± 8.4	0.051	4 [3–7] vs. 4 [3–7]	0.203	9.5% vs. 9.2%	0.873
Adenovirus	1265	629	636	17.0 ± 17.3 vs. 16.1 ± 7.7	0.578	6.5 ± 6.8 vs. 5.9 ± 7.4	0.002	4 [3–6] vs. 4 [3–6]	0.008	5.7% vs. 7.5%	0.234
Influenza A	533	440	93	10.1 ± 6.3 vs. 12.4 ± 7.6	**0.002**	3.9 ± 6.0 vs. 4.3 ± 6.0	0.322	4 [3–5] vs. 4 [3–5]	0.980	4.3% vs. 7.5%	0.298
Influenza B	163	134	29	9.7 ± 6.2 vs. 12.1 ± 5.7	**0.019**	3.7 ± 6.0 vs. 5.3 ± 6.4	**0.019**	4 [3–5] vs. 5 [4–7]	**0.028**	6.7% vs. 13.8%	0.370
Parainfluenza	531	330	201	14.2 ± 15.9 vs. 15.3 ± 14.8	0.129	4.7 ± 6.7 vs. 5.8 ± 8.1	0.076	4 [3–7] vs. 4 [3–6]	0.542	7.6% vs. 5.5%	0.449
HMPV	410	226	184	12.2 ± 6.5 vs. 15.2 ± 8.1	** < 0.001**	5.3 ± 6.3 vs. 6.3 ± 9.2	0.503	5 [3–6] vs. 4 [3–6]	0.520	5.3% vs. 4.9%	1.000
SARS-CoV-2	382	343	39	10.5 ± 6.3 vs. 12.3 ± 4.9	**0.014**	3.5 ± 6.7 vs. 5.6 ± 14.9	0.066	4 [2–6] vs. 5 [4–8.5]	**0.003**	8.7% vs. 25.6%	**0.003**

Each viral subgroup compares patients with a single detected virus (e.g., RSV only) to those with the same virus plus ≥ 1 additional virus. Continuous variables were compared using the Wilcoxon rank-sum test, and categorical variables were compared using the chi-square test. Abbreviations: *WBC* White blood cell count, *CRP* C-reactive protein, *LOS* Length of stay, *ICU* intensive care unit, *RSV* respiratory syncytial virus, *HMPV* human metapneumovirus, *SARS-CoV-2* severe acute respiratory syndrome coronavirus 2

## Discussion

In this large retrospective study of 5,703 hospitalized children with respiratory viral infections, we found that approximately one in five patients (19.6%) had co-infections with two or more viruses. Children with multiple viral infections were younger compared with those with single-virus infections, a finding consistent with previous reports that co-infections occur most frequently in infants and young children [10–12]. This pattern has been attributed in previous studies to immature immune systems, limited protective immunity from prior viral exposures, and prolonged viral shedding in younger children compared with older children and adults [10]. Rhinovirus was the most commonly detected pathogen among co-infected patients, again in line with prior studies [13].

A key finding of our study was that children with multiple viral infections were more likely to present with higher inflammatory and hematologic indices, including modestly elevated CRP, WBC, neutrophil, and lymphocyte counts. This observation contrasts with several previous studies that reported no significant association between viral co-infection and inflammatory marker levels [14–16]. Our results therefore suggest that multiple viral infections may, in some settings, elicit a stronger systemic inflammatory response than previously recognized. This phenomenon was particularly robust in specific viral subgroups, such as RSV or Influenza B, where co-infection was associated with significantly higher inflammatory markers. The higher rate of chest radiography observed among co-infected children may reflect increased diagnostic evaluation in this population.

Elevated inflammatory markers are often interpreted as suggestive of bacterial infection [17, 18], which can lead clinicians to initiate antibiotic therapy. The observation that multiple viral infections are independently associated with higher inflammatory markers highlights the potential for diagnostic confusion and unnecessary antibiotic use. Although antibiotic exposure did not differ between groups in our cohort, children with multiple viral infections were more frequently treated with bronchodilators as well as inhaled and systemic corticosteroids, suggesting a slightly increased need for anti-inflammatory and supportive therapies.

Despite these laboratory differences, the overall clinical severity of illness was not significantly different between groups. Length of stay, ICU admission, and rehospitalization rates did not differ significantly, consistent with findings from high-quality systematic reviews and meta-analyses, such as the large systematic review by Scotta et al., which found no significant differences between single and multiple viral infections in length of stay, ICU admissions, mechanical ventilation requirements, or mortality [19]. Taken together, these findings suggest that viral co-infections may provoke a stronger inflammatory response without translating into worse clinical outcomes. While primary clinical outcomes such as length of stay and ICU admission rates remain consistent with prior reports, our findings demonstrate that viral co-infection impacts the systemic inflammatory response and the intensity of medical management, including increased diagnostic imaging and pharmacological interventions.

Strengths of this study include the large sample size and inclusion of a diverse spectrum of pediatric age groups, enhancing the generalizability of the findings. The use of multiplex PCR panels allowed for comprehensive and sensitive viral detection across a wide range of respiratory pathogens. Disease severity was assessed across multiple dimensions, including hospitalization parameters, clinical signs, laboratory indices, and medication use, providing a thorough evaluation of clinical impact. These findings provide a reliable basis for future prospective investigations. Furthermore, the extended study period provides a broader temporal context for interpreting the findings.

Limitations include its retrospective design, missing data for some laboratory and clinical parameters, and inability to fully assess long-term or outpatient outcomes. Additionally, frequent changes in viral sampling protocols, particularly during the COVID-19 pandemic, may have affected the detection rates of certain viruses and the consistency of co-infection reporting. The seven-year duration of this study (2017–2024) necessitated the use of different multiplex PCR diagnostic platforms as laboratory protocols evolved. While all platforms utilized were high-sensitivity assays targeting the same core respiratory pathogens, subtle differences in diagnostic thresholds or sensitivity for specific viral targets may have existed. For example, SARS-CoV-2 was not included in the diagnostic panels prior to the COVID-19 pandemic. Similarly, testing for rhinovirus was only integrated into the diagnostic panels starting in 2020. To address potential longitudinal bias, we performed a validation analysis by repeating the primary comparisons while omitting rhinovirus detections from the analysis. As the results were highly consistent with our main findings, it suggested that the delayed inclusion of this target did not alter the study's overall conclusions. Furthermore, as the respiratory panels used detect only viral pathogens, this study lacks specific data on viral-bacterial co-infections. Finally, it should be noted that due to the extensive number of clinical and laboratory parameters analyzed in this study, formal adjustments for multiple comparisons were not performed. Consequently, our findings should be interpreted as exploratory. Nevertheless, the consistency of the results across related clinical outcomes suggests that these findings reflect meaningful patterns that warrant further validation in future prospective trials.

Overall, our findings suggest that viral co-infections in children are common and associated with a modestly heightened inflammatory response, but do not appear to substantially worsen clinical outcomes. Future prospective studies are needed to clarify the mechanisms behind co-infection responses, evaluate preventive strategies specifically for younger populations, and guide targeted management strategies.

## Data Availability

The anonymized data that support the findings of this study are available from the corresponding author upon reasonable request.
